# Bupropion, methylphenidate, and 3,4-methylenedioxypyrovalerone antagonize methamphetamine-induced efflux of dopamine according to their potencies as dopamine uptake inhibitors: implications for the treatment of methamphetamine dependence

**DOI:** 10.1186/1756-0500-6-220

**Published:** 2013-06-05

**Authors:** Linda D Simmler, Rebecca Wandeler, Matthias E Liechti

**Affiliations:** 1 Psychopharmacology Research Group, Division of Clinical Pharmacology and Toxicology, Department of Biomedicine and Department of Internal Medicine, University Hospital Basel and University of Basel, Hebelstrasse 2, Basel CH-4031, Switzerland

**Keywords:** Methamphetamine, Addiction, Dopamine, Dopamine transporter, Bupropion, Methylphenidate, MDPV

## Abstract

**Background:**

Methamphetamine-abuse is a worldwide health problem for which no effective therapy is available. Inhibition of methamphetamine-induced transporter-mediated dopamine (DA) release could be a useful approach to treat methamphetamine-addiction. We assessed the potencies of bupropion, methylphenidate, and 3,4-methylenedioxypyrovalerone (MDPV) to block DA uptake or to inhibit methamphetamine-induced DA release in HEK-293 cells expressing the human DA transporter.

**Findings:**

Bupropion, methylphenidate, and MDPV inhibited methamphetamine-induced DA release with relative potencies corresponding to their potencies to block DA uptake (potency ranks: MDPV > methylphenidate > bupropion).

**Conclusions:**

Bupropion and methylphenidate antagonize the effects of methamphetamine in vitro and may be potential candidates for the treatment of stimulant addiction. However, drugs that very potently antagonize the effect of methamphetamine are likely to also exhibit considerable abuse liability (MDPV > methylphenidate > bupropion).

## Findings

### Background

Methamphetamine dependence is a major public health problem. Currently, no medical treatments are approved for stimulant dependence indicating the need to explore potential candidates [1]. Methamphetamine releases dopamine (DA) via the DA transporter (DAT) [2,3]. DA is thought to mediate the reinforcing effects of psychostimulants, which lead to drug dependence [4,5]. Blocking the pronounced release of DA by methamphetamine may therefore be an interesting therapeutic option for the treatment of methamphetamine dependence [1]. Bupropion and methylphenidate are DA uptake inhibitors that interact with the same pharmacological target as methamphetamine [6-11]. Bupropion is used as an antidepressant and smoking cessation aid [7,9]. Methylphenidate is effectively used in the treatment of attention-deficit/hyperactivity disorder [12,13]. In addition, small clinical studies indicated promising beneficial effects for both medications in methamphetamine dependence [1]. Bupropion reduced the acute subjective effects of methamphetamine in a laboratory study [14] and methamphetamine use in dependent patients with moderate drug use [15-18]. Methylphenidate reduced amphetamine use in dependent patients [19] and it is now being investigated in methamphetamine-addiction (clinicaltrials.gov: NCT01044238). Bupropion also reduced methamphetamine self-administration in rats [20] or rhesus monkeys [21]. In contrast, methylphenidate did not affect methamphetamine self-administration in rhesus monkeys [21].

The precise pharmacological mechanism of action of bupropion and methylphenidate with regard to their therapeutic effects in methamphetamine dependent patients is not known. Dopamine is thought to contribute to the drug-high and euphoria produced by psychostimulants and mediates the addictive properties of drugs of abuse [4,22]. Amphetamines reverse the transport of DA through the DAT and this effect is thought to play a key role in the addictive potential of amphetamines [5]. The DA uptake inhibitors bupropion and methylphenidate may therefore prevent methamphetamine from interacting with the DAT to release DA, and such an effect would antagonize effects of methamphetamine. Several DA uptake inhibitors have previously been shown to prevent DAT-mediated release of DA by amphetamines in vitro. For example, bupropion and methylphenidate [23] as well as GBR12909 [3] inhibited DAT-mediated amphetamine- or methamphetamine induced DA release from rat synaptosomes. In HEK-293 cells expressing human DAT, methylphenidate inhibited DA efflux induced by methamphetamine [24]. These and similar data suggest that bupropion and methylphenidate block the interaction of methamphetamine with the DAT to release DA and thereby act as antagonists of amphetamine-like drugs.

The aim of the present study was to test and compare the effects of bupropion and methylphenidate on methamphetamine-induced DA efflux in HEK-293 cells expressing human DAT in vitro. Bupropion and methylphenidate were selected because of their availability as licensed medications and the clinical data described above. We also included 3,4-methylenedioxypyrovalerone (MDPV) into the study because it has been shown to be a very potent DAT inhibitor [10,25].

We hypothesized that 1) the DA uptake blockers would prevent methamphetamine-induced DA release and 2) the potencies of the drugs to inhibit methamphetamine-induced DA release would correspond to their potencies to block DA uptake.

### Methods

#### Drugs

(±)-Bupropion hydrochloride was from Toronto Research Chemicals (North York, Canada), d-methamphetamine, (±)-methylphenidate, and (±)-MDPV were supplied as hydrochloride salts by Lipomed (Arlesheim, Switzerland).

#### Inhibition of DA uptake

The potencies of the drugs to inhibit the DAT were evaluated as previously described [26] in HEK-293 cells (Invitrogen, Zug, Switzerland) stably transfected with the human DAT [8].

#### Inhibition of methamphetamine-induced DA release

We performed DA transporter mediated release experiments as previously published [25] with slight modification. In brief, HEK-293 cells expressing the human DAT as stated above were cultured in 48 well-plates. Cells were filled with ^3^H-DA, washed, and incubated with 250 μL buffer containing the drug alone or in combinations. Drug combinations were 10 μM of methamphetamine with bupropion, methamphetamine, or MDPV in different concentrations. DA release was stopped after 15 min by removing the release buffer from the cells. To quantify the DA release we determined the radioactivity in the cells after another wash step. The residual radioactivity in the cells after methamphetamine alone defined 100% DA release. Baseline (0% release) was defined as the radioactivity remaining in the cells treated with bupropion, methylphenidate, or MDPV alone at the highest concentration used.

### Results

#### Inhibition of DA uptake

**Figure 1:**
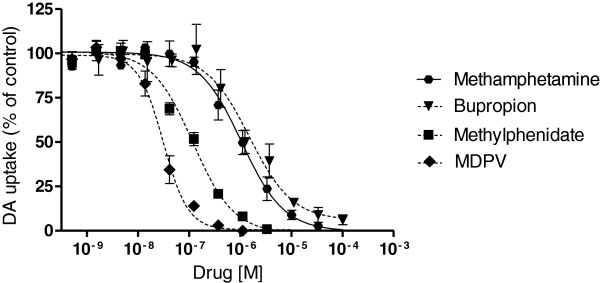
**DA uptake inhibition by methamphetamine, bupropion, methylphenidate, and MDPV.** IC_50_ values are shown in Table 1. The data are expressed as mean ± SEM of 3–4 independent experiments.

Bupropion, methylphenidate, and MDPV inhibited the uptake of DA. MDPV was the most potent DAT inhibitor followed by methylphenidate and bupropion. Methamphetamine blocked DA uptake with similar potency to bupropion (Figure 1).

#### Inhibition of methamphetamine-induced DA release

**Figure 2:**
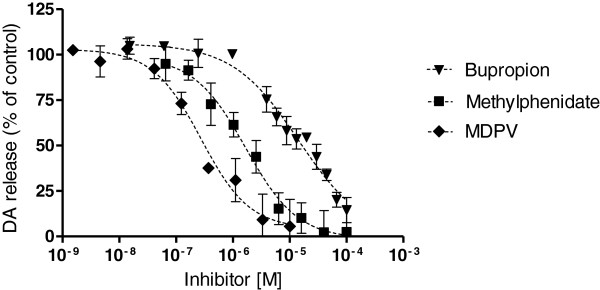
**Methamphetamine-induced DA release is inhibited by bupropion, methylphenidate and MDPV.** The corresponding IC_50_ values are shown in Table 1. DA release was induced with 10 μM methamphetamine (100% release) and blocked with different concentration of the inhibitors bupropion, methylphenidate or MDPV. Baseline (0% release) was defined as the radioactivity remaining in the cells treated with bupropion, methylphenidate, or MDPV alone. The data are expressed as mean ± SEM of 3–4 independent experiments.

**Figure 3:**
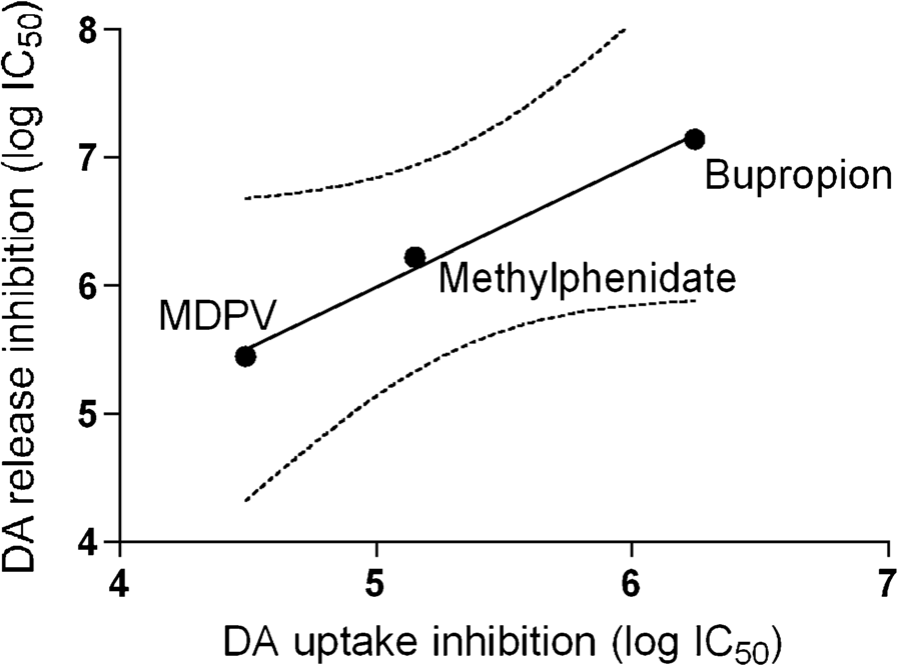
**The potencies of the drugs (log IC**_**50 **_**values) to inhibit DA uptake correlate linearly with their potencies to inhibit methamphetamine-induced DA release (correlation coefficient > 0.99, p < 0.05, regression line and 95% confidence intervals).**

Methamphetamine released DA with a potency (EC_50_) of 1.56 μM (0.9 μM-2.8 μM, 95% CI) as shown previously [25]. DA release induced with 10 μM methamphetamine was inhibited concentration-dependently by bupropion, methylphenidate, and MDPV (Figure 2). MDPV was the most potent inhibitor of the methamphetamine-induced DA release followed by methylphenidate and bupropion (Figure 2). The IC_50_ values are shown in Table 1. The potencies (IC_50_ values) of the drugs to block DA release correlated highly with the potencies to block DA uptake (Figure 3) as confirmed by a correlation coefficient of >0.99, p < 0.05.

**Table 1:** Potencies of drugs to block DA uptake or methamphetamine-induced DA release

	**DA uptake**	**Methamphetamine-induced DA release**
	IC_50_ (μM) (95% CI)	IC_50_ (μM) (95% CI)
Methamphetamine	1.05 (0.7-1.5)	-
Bupropion	1.76 (1.1-2.8)	14.2 (9.7-21)
Methylphenidate	0.14 (0.1-0.2)	1.67 (0.7-4.0)
MDPV	0.031(0.03-0.04)	0.28 (0.1-0.6)

Values are means of 3–4 independent experiments and 95% confidence intervals (CI).

### Discussion

In the present study, the DA uptake inhibitor bupropion inhibited DA release induced by methamphetamine. This mechanism might underlie the reduction in the methamphetamine-induced subjective drug high by bupropion pretreatment documented in a clinical laboratory study [14] and the reduced methamphetamine consumption in drug users treated with bupropion [15-18]. Methylphenidate also blocked the methamphetamine-induced DA release similar to bupropion and this effect may also antagonize the rewarding effects of methamphetamine and its use in dependent patients. In fact, methylphenidate showed beneficial effects in amphetamine dependent patients [19] and is being investigated for the treatment of methamphetamine addiction (clinicaltrials.gov: NCT01044238). Thus, inhibition of DA release might be a pharmacological mechanism how DA uptake inhibitors reduced the subjective stimulant drug effects or drug consumption in the clinical studies noted above. In addition, methylphenidate and bupropion also increase DA levels and therefore both drugs may also act as substitution treatments for methamphetamine use. In the present study we also included the very potent DA uptake inhibitor MDPV to explore how the potency of a drug as DA uptake inhibitor relates to its potency to antagonize the pharmacological effect of methamphetamine. MDPV blocked methamphetamine-induced DA release with high potency reflecting its high potency as an uptake inhibitor. We found that the potencies of the drugs to block methamphetamine-induced DA release correlated closely and significantly with their potencies to act as DA uptake inhibitors. The finding suggests that the more potent a drug antagonizes the DA release produced by methamphetamine the more potently it also blocks DA uptake. This finding may have important clinical implications regarding the abuse liability of potential antagonist treatments for methamphetamine dependence. With regard to the drugs tested in the present study, the antidepressant bupropion is a low-potency DA transporter inhibitor and it is considered a drug that does not produce relevant euphoria nor addiction [27,28]. Methylphenidate is an intermediate-potency DA transporter inhibitor and is known to produce euphoria at higher doses [29,30] and to have a relevant abuse potential [31,32]. The cathinone MDPV is a high potency DA transporter inhibitor and street designer drug (“super coke”, “research chemical”, “bath salt”) with high addiction potential similar to the DA releaser methamphetamine [25,33-35]. Our findings indicate that drugs that potently and effectively antagonize the effect of methamphetamine are likely to exhibit high abuse liability themselves because they block DA uptake. In fact, the potency of amphetamine-type stimulants to block DA uptake has been shown to correlate with the doses used by humans [25]. Furthermore, potent DA transport uptake inhibition is sufficient to produce addiction because cocaine and MDPV only block DA uptake and do not induce DA release as methamphetamine [5,25]. It is therefore questionable whether there are any compounds that do not activate the DA system and lack abuse liability but effectively prevent methamphetamine from interacting with DAT. On the other hand, abuse liability of medications can be reduced by using extended-release drug formulations.

Methamphetamine also has additional effects on the DA system (e.g., on monoamine oxidase and the vesicular monoamine transporter), which were not studied here. These effects of methamphetamine take place within the cells and are likely prevented by DAT inhibitors [36] that block methamphetamine transport into the cell. Methamphetamine also releases norepinephrine [2,3] and norepinephrine is thought to contribute to the acute effects of amphetamine-type drugs [3,37-39]. MDPV [25] and methylphenidate [6,40], and to a lower extent bupropion [7,11], block the norepinephrine transporter and these drugs could also block methamphetamine-induced norepinephrine release. We did not address potential drug interactions at the norepinephrine transporter because in contrast to DA, norepinephrine is not generally thought to be a major mediator of the addictive properties of psychostimulants. However, interactions at the norepinephrine transporter could be expected to contribute to any therapeutic effects of the drugs tested in the present study. Finally, it should be noted that we assessed only a small number of DAT inhibitors. However, the drugs were selected to cover a wide range of DAT inhibition potencies including also the very potent DAT inhibitor MDPV.

### Conclusion

Our in vitro studies and the limited clinical data indicate that the low- and intermediate-potency DA uptake inhibitors bupropion and methylphenidate may be potential candidates as treatments of amphetamine-type stimulant dependence [1] due to their property to inhibit methamphetamine-induced DA efflux. Their clinical efficacy needs further confirmation.

### Availability of supporting data

The data supporting the results of this article are included within the article. This work was supported by the Swiss National Science Foundation (grant no. 32323B_144996). Publication costs are supported by the Neurex network (http://www.neurex.org).

## Abbreviations

DA: Dopamine; DAT: Dopamine transporter; MDPV: 3,4-methylenedioxypyrovalerone.

## Competing interests

The authors declare that they have no competing interests.

## Authors’ contributions

LDS and MEL designed this study and wrote the manuscript. RW and LDS performed the experiments. All authors analyzed the data. All authors have read and approved the final manuscript.
